# A Journey from Thermally Tunable Synthesis to Spectroscopy of Phenylmethanimine in Gas Phase and Solution

**DOI:** 10.1002/chem.202003270

**Published:** 2020-10-15

**Authors:** Alessio Melli, Simone Potenti, Mattia Melosso, Sven Herbers, Lorenzo Spada, Andrea Gualandi, Kevin G. Lengsfeld, Luca Dore, Philipp Buschmann, Pier Giorgio Cozzi, Jens‐Uwe Grabow, Vincenzo Barone, Cristina Puzzarini

**Affiliations:** ^1^ Scuola Normale Superiore Piazza dei Cavalieri 7 56126 Pisa Italy; ^2^ Dipartimento di Chimica “Giacomo Ciamician” Universitá di Bologna Via Selmi 2 40126 Bologna Italy; ^3^ Institut für Physikalische Chemie und Elektrochemie Gottfried Wilhelm Leibniz Universität Hannover Callinstraße 3A 30167 Hannover Germany

**Keywords:** astrochemistry, computational chemistry, NMR spectroscopy, rotational spectroscopy

## Abstract

Phenylmethanimine is an aromatic imine with a twofold relevance in chemistry: organic synthesis and astrochemistry. To tackle both aspects, a multidisciplinary strategy has been exploited and a new, easily accessible synthetic approach to generate stable imine‐intermediates in the gas phase and in solution has been introduced. The combination of this formation pathway, based on the thermal decomposition of hydrobenzamide, with a state‐of‐the‐art computational characterization of phenylmethanimine laid the foundation for its first laboratory observation by means of rotational electric resonance spectroscopy. Both *E* and *Z* isomers have been accurately characterized, thus providing a reliable basis to guide future astronomical observations. A further characterization has been carried out by nuclear magnetic resonance spectroscopy, showing the feasibility of this synthetic approach in solution. The temperature dependence as well as possible mechanisms of the thermolysis process have been examined.

## Graphical abstract

**A multidisciplinary approach** involving organic synthesis and high‐resolution molecular spectroscopy has led to the definition of a thermally tunable formation route for imines. Its application to phenylmethanimine combined with an integrated rotational spectroscopy–quantum chemistry protocol has enabled the full characterization of the latter, thus providing the means for future astronomical observations.

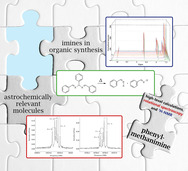

## Introduction

In the last few decades, astrochemistry—the research field focused on the chemical composition and evolution of ordinary matter in space—has flourished dynamically: born as a niche sector, it grew up into a field of broad interest. Its birth is relatively recent because, for many years, the interstellar medium (ISM)—the space between star systems in a galaxy—has been considered too hostile to bear any chemical complexity. However, with the rise of radio astronomy (in the 1960s), it became evident that the ISM harbors a diverse collection of interesting polyatomic (both organic and inorganic) molecules.[^1^, ^2^] Since then, a large number of molecular species has been detected.^[3]^ Among them, those having a prebiotic character are of particular interest because of their key role in postulated mechanisms leading to the emergency of life. Imines belong to this category.

While their astrobiological relevance has not yet unambiguously proven, imines are known to play major roles in many chemical processes. They have been recognized as crucial intermediates in organic synthesis, due to their extensive use in the preparation of N‐containing compounds,^[4]^ and as naturally or biologically active molecules.^[5]^ Because of peculiar and extreme conditions, the chemical reactivity in space is very different from the terrestrial counterpart, and unstable species (even ions and radicals) can survive long. As a consequence, reactive species such as some N−H imines, whose isolation is difficult on Earth, can be observed in the ISM,[^6^, ^7^, ^8^, ^9^, ^10^] with the prerequisite of a successful laboratory spectroscopy characterization. With the chemistry in the ISM being new and intriguing, severe difficulties arise when interstellar molecules are to be produced by “terrestrial” organic procedures for their characterization, e.g., by molecular spectroscopy. Indeed, the instability of these species often requires that they are directly generated inside the spectrometer using harsh conditions and rather poorly predictable techniques such as pyrolysis or electric discharge. We have tried to overcome these difficulties by developing a different and easier synthetic route, the thermally tunable formation of imines, which has been tested both in the gas phase and in solution.

The subject of this work is the case study of phenylmethanimine (PMI), which is a non‐standard reactive molecule of potential astrochemical relevance. Due to the large amount of hydrogen (in the form of H, H_2_, and also H^+^/H_3_^+^) in the ISM, hydrogenation is an efficient process^[11]^ and leads to the formation of saturated or partially saturated molecules.^[12]^ On this ground, hydrogenation of benzonitrile (BN) in the ISM can yield PMI, an interesting imine in terms of chemical complexity and molecular evolution. This gained attention because of the recent detection of BN in the cold‐core Taurus Molecular Cloud 1 (TMC‐1), which provided the unequivocal proof of the presence of benzene in that cold environment, and—more generally—in the ISM.^[13]^ In turn, benzene is the building block of polycyclic aromatic hydrocarbons (PAHs), which are recognized (but spectroscopically not proven) to be important constituents of small dust grains in interstellar clouds and are believed to play a key role in the chemical evolution in space from both a catalytic and protective point of view.^[14]^

Despite its appeal as a good approach for imine synthesis in the ISM, on Earth, nitrile reduction using molecular hydrogen usually requires catalytic conditions which are not compatible with a single hydrogenation process. Catalytic processes directly lead to the saturated derivative, that is, the corresponding amine, or to a mixture of primary‐, secondary‐ and even tertiary‐amines via reaction of the N−H imine intermediates.[^15^, ^16^] This is due to the high reactivity of the imine intermediate.[^17^, ^18^, ^19^, ^20^, ^21^] The N−H imine derivatives of aliphatic or aromatic aldehydes are known to be much less stable, and only in a few cases they have been successfully isolated or characterized.[^22^, ^23^] Indeed, the literature is rich of examples supporting the instability and reactivity of these imines. The chemical labile nature of N−H imines, and the difficulties related to their isolation, resulted in the employment of imines possessing various substituents on the nitrogen atom (N‐substituted imines). Therefore, the activating or protecting groups introduced on the nitrogen atom need to be removed after the synthetic endeavor. Based on the concept of atom economy, N‐unsubstituted imines would be ideal, since deprotection steps, to obtain the final products, are not required. Unfortunately, unlike N‐substituted imines, the approaches to N‐unsubstituted imines are limited to (and, in many cases, were proposed as) unstable, not characterized intermediates.^[24]^ The direct reaction with ammonia and aldehydes is not a suitable synthetic procedure for such imines. N−H imines can be accessed by the controlled hydrolysis of N‐metalloimines or silylimines.^[25]^ Another simple access to N−H imines involves available azides precursors with the removal of N_2_ and subsequent migration of hydrogen, under catalytic conditions.^[26]^ Nevertheless, all these approaches are impracticable in order to generate N−H imines for rotational spectroscopy investigations in gas phase. Indeed, such context increased the relevance of a thermally tunable approach from a simple precursor for the generation of N−H imines and their spectroscopic investigation. Such approaches avoid critical issues such as: (i) demanding experimental setups, that is, subtle control of reaction conditions; (ii) trapping procedures leading to imine complexes; (iii) use of transition metal catalysts; (iv) unfeasible interface with spectroscopic techniques.

The last aspect is of particular interest to our investigation since, in order to set up and validate our synthetic route, high‐resolution spectroscopic techniques offer the highest specificity: rotational electric resonance (RER) spectroscopy in the gas phase and nuclear magnetic resonance (NMR) spectroscopy in solution. Since our strategy is based on two key points, namely (i) the use of stable and affordable organic precursors and (ii) the generation of the unstable and reactive imines through simple conditions, it provides a suitable way to produce imines directly inside the spectrometer.

In this manuscript, we report a full account of our successful endeavor, which has led ‐for the first time‐ to a complete and accurate spectroscopic characterization of PMI using RER, which is prerequisite for its identification in the ISM by means of radio astronomy. It required a multidisciplinary effort combining organic synthesis and molecular spectroscopy, supported and guided by computational chemistry.

## Results and Discussion

### Spectroscopic characterization of PMI

**Figure 1:**
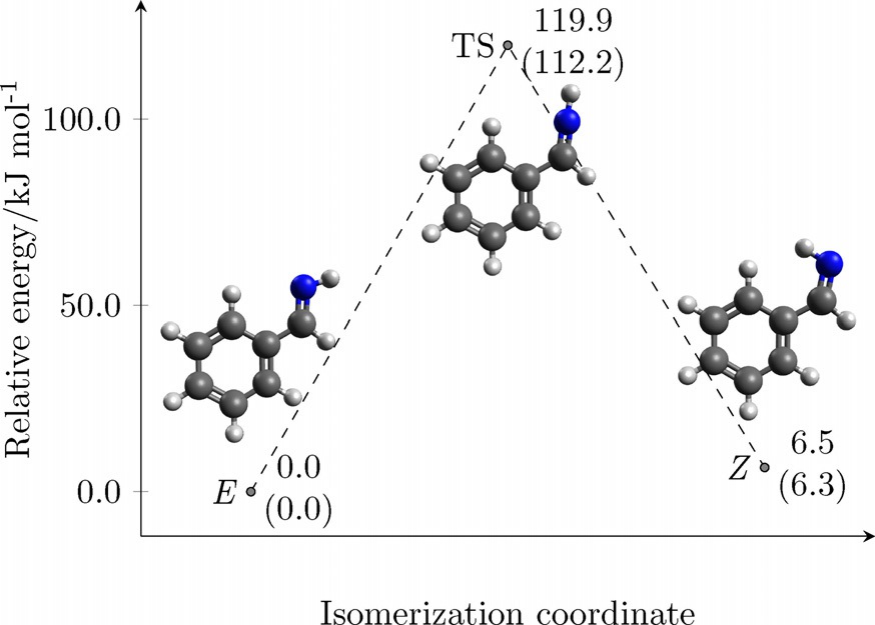
Stationary points of PMI, relative electronic energies at the ChS level. Values within parentheses are augmented by harmonic B2PLYP zero‐point energy corrections.

To guide RER experiments, a state‐of‐the‐art quantum‐chemical (QC) characterization has been performed. Using the density functional theory (DFT, see Supporting Information, for details), a preliminary scan of the potential energy surface has been carried out, which located two isomers (*E* and *Z*, referring to the relative position of the phenyl group with respect to the imine hydrogen, see Figure 1). Subsequently, an accurate structural determination has been obtained by resorting to the so‐called “cheap” composite scheme^[27]^ (hereafter denoted as ChS), whose denomination refers to the limited computational cost in spite of its accuracy.

The ChS approach is expected to provide results with an accuracy of about 0.001–0.002 Å for bond lengths and 0.1–0.2° for angles.^[27]^ This approach has also been used to derive accurate electronic energies, which are given in Figure 1. While structural parameters and a detailed description of the ChS model are provided in the Supporting Information, this Figure points out that the *E* isomer is about 6 kJ mol^−1^ more stable than the *Z* species. TS_E‐*Z*_ has a quasi‐linear C‐N‐H geometry, with the isomerization process occurring in the molecular plane, similarly to what already observed for other imines such as ethanimine (ETI)^[28]^ and C‐cyanomethanimine (CCMI).^[29]^ Although different computational schemes have been applied, a qualitative comparison between the relative energies is possible. A smaller energy difference between the two isomers was obtained for ETI and CCMI (2.8 kJ mol^−1^ and 2.0 kJ vmol^−1^, respectively[^28^, ^29^]), while a similar isomerization barrier was found (about 116 kJ mol^−1^ for ETI,^[28]^ ∼111 kJ mol^−1^ for CCMI,^[29]^ and about 112 kJ mol^−1^ for PMI).

Equilibrium rotational constants have been straightforwardly derived from the ChS equilibrium structure, while the required vibrational corrections have been evaluated from DFT anharmonic force‐field calculations (thereby using B3LYP,[^30^, ^31^] see Supporting Information for details). First‐order properties, such as dipole moment components and nuclear quadrupole coupling constants, have been computed using, in the framework of DFT, the double‐hybrid B2PLYP functional^[32]^ (see Supporting Information for details). This level of theory has also been employed for deriving centrifugal distortion constants.

The gas‐phase characterization of PMI has been performed in the 3–26 GHz range, using the COBRA‐type (Coaxially Aligned Beam Resonator Arrangement) Fourier Transform Microwave Spectrometer (FTMW), described in details elsewhere.^[33]^ A solenoid valve with reservoir, filled with hydrobenzamide (HBA) and heated to 85 °C, was used to vaporize it and, at the same time, to thermally‐decompose it, thus producing PMI in the gas phase. HBA was synthesized, following the procedure described in literature,[^17^, ^34^, ^35^, ^36^] by condensation of benzaldehyde and ammonium hydroxide solution and obtained as white solid, with its identity and purity being confirmed by NMR analysis (see the Supporting Information for details).

**Figure 2:**
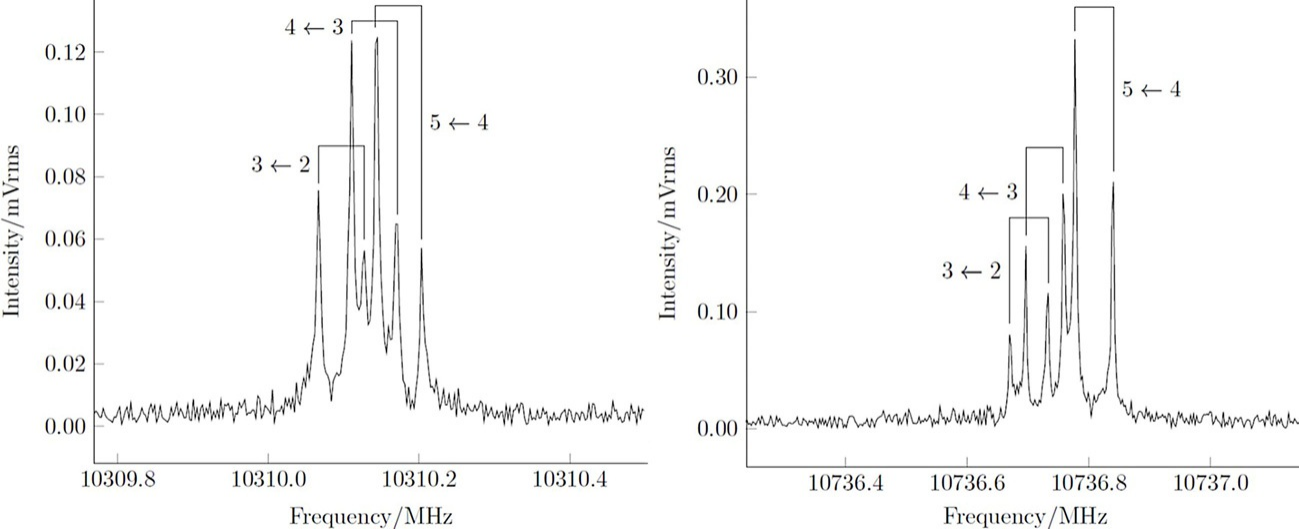
The 4_1,4_←3_1,3_ transition of *E*‐phenylmethanimine (left panel) and the 4_0.4_←3_0.3_ transition of the *Z* isomer (right panel), denoting rotational transitions *J*_*Ka*,*Kc*_ *←J’*_*Ka*’,*Kc*’_. In both spectra, the reported quantum numbers refer to the nuclear quadrupole coupling Scheme *F*←*F*’, *F*=*J*+*I* and the line pairs becoming split by the Doppler effect are made evident.

Given the high accuracy of the QC calculations performed (the relevant spectroscopic parameters are collected in Table 1), the computational prediction of the rotational spectrum was expected to match the accuracy^[37]^ required for an unequivocal identification of the *E* and *Z* isomers of phenylmethanimine in the gas phase. Indeed, it was straightforward to successfully identify and assign more than one hundred transition frequencies for both PMI isomers. Their unique pattern (hyperfine structure), due to quadrupole coupling splitting originated by the presence of the ^14^ n nucleus (*I*=1), is shown in Figure 2 for both PMI isomers. Furthermore, we note that each rotational transition appears as a doublet due to the Doppler effect arising from a supersonic jet expansion. For the most stable *E*‐PMI, the spectral analysis has been extended in the 83–100 GHz range, thus allowing the improvement of the spectroscopic parameters, using a frequency‐modulation millimeter‐wave (FM‐mmW) spectrometer (see ref. [38] for a detailed description). In this experiment, a different production method of PMI has been employed, as reasoned below. Indeed, PMI was produced by means of flash vacuum pyrolysis (FVP)[^39^, ^40^] of a sample of *α*‐methylbenzylamine (890 °C).

**Table 1:** Comparison between experimental (exp.)^[a]^ and calculated (best theo.) spectroscopic parameters (expressed in MHz, dipole moment components are given in debye).

	*E*‐phenylmethanimine		*Z*‐phenylmethanimine	
	exp.	best theo.	exp.	best theo.
*A*	5217.29202(11)	5216.95	5200.81278(16)	5200.42
*B*	1565.283633(28)	1564.68	1548.969349(92)	1548.76
*C*	1204.540307(14)	1204.05	1194.842313(78)	1194.30
*D_J_*×10^5^	5.775(11)	5.57	5.643(35)	5.28
*D_K_*×10^4^	7.65(11)	7.07	7.06^[b]^	7.06
*D_JK_*×10^4^	1.678(37)	1.81	1.445(47)	1.57
*d*_1_×10^5^	−1.6941(55)	−1.63	−1.542(44)	−1.51
*d*_2_×10^6^	−3.162(23)	−3.00	−2.68^[b]^	−2.68
1.5χ_aa_	1.5271(14)	1.61	−5.8871(23)	−6.05
0.25(χ_aa_−χ_cc_)	−1.70630(38)	−1.83	−0.4954(15)	−0.55
|μ_a_|		0.77		3.09
|μ_b_|		1.66		0.15
|μ_c_|		0		0
# lines^[c]^	180		118	
rms x 10^3^	4.56		1.38	
*σ* ^[d]^	0.65		0.69

[a] Watson's S‐reduction, *I* ^r^ representation. Standard errors are reported within parentheses. [b] Fixed at the corresponding computed value. [c] Distinct frequency lines. [d] Dimensionless quantities.

The spectroscopic parameters derived from a weighted non‐linear fit of the observed transitions, performed using the Pickett's CALPGM suite of programs,^[41]^ are reported in Table 1. An excellent agreement between the computed and experimental values can be noted, with an average error of 0.03 % on the effective rotational constants and a maximum discrepancy below 0.05 %. Only small deviations have also been found for the quartic centrifugal distortion and nuclear quadrupole coupling constants. Since rotational and quadrupole coupling constants are strongly tied to the molecular structure, the very good agreement gives further confidence in the reliability and accuracy of the computed geometries.

While the full list of the rotational transitions included in the fit is given in the Supporting Information, we state here that the root mean square error (rms) is smaller than 5 kHz, with a standard deviation (*σ*) slightly below unity, that is, achieving experimental accuracy.

### Thermal decomposition of HBA

The successful generation and characterization of PMI in the gas phase raised questions about the mechanism of its formation and the possible extension of the methodology to the condensed phase (i.e. solution) and, more generally, to other N‐unsubstituted imines (thus avoiding more expensive and/or more chemically demanding procedures). For this reason, the thermal behavior of HBA has been explored from room temperature to 100–110 °C, both in the gas phase by means of RER and in solution by ^1^H‐NMR.

In particular, the *J*_*Ka,Kc*_=5_0,5_←4_0,4_ rotational transition of four molecular species, namely BA, BN, and the *E* and *Z* isomers of PMI, has been recorded with the COBRA‐FTMW spectrometer. To provide a reliable comparison between the intensity of the transitions, the field amplitude of the microwave excitation pulse has been scaled reciprocal to the value of dipole moment component along the *a*‐axis and a 2000 averaging signal acquisition has been chosen.

**Figure 3:**
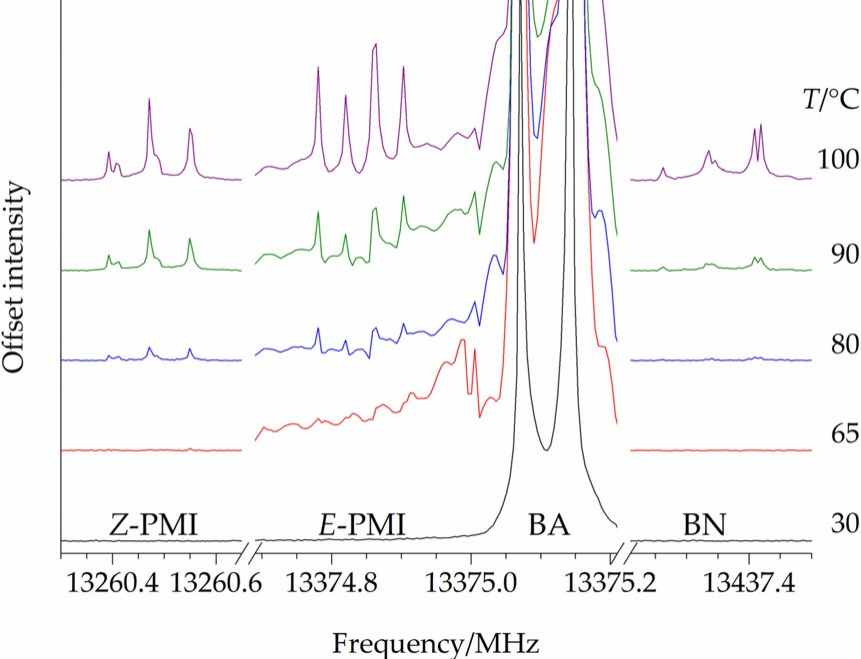
Spectra of the *J*_*Ka*,*Kc*_=5_0,5_←4_0,4_ transition for the four molecular species investigated (PMI: phenylmethanimine; BA: benzaldehyde, BN: benzonitrile). The huge abundance of BA in the gas phase leads to out‐of‐chart transitions at any temperature.

The recording has been started ten minutes after the temperature set up. The recorded spectra (see Figure 3) reveal the presence of BA at all temperatures, while clearly distinguishable signals of PMI isomers are observed only above 80 °C. BN lines appear at the same temperature. For both PMI isomers and BN, it is noted that rising the temperature increases the intensity of the transitions, thus improving the signal‐to‐noise ratio (S/N). Although not shown in Figure 3, the 12 321.0 MHz transition of the water dimer^[42]^ was observed for each temperature increment. This confirms the steady presence of water in the experimental apparatus, which very likely plays a key role in the investigated hydrolytic process.

An important note on the relative populations of the two PMI isomers is deserved. In principle, this information should be derivable from the spectra of Figure 3; however, the *E*‐PMI transition reported there lies very close to the strong transition of BA, thus clearly modifying the baseline of the *E*‐PMI transition. As a consequence, the signal‐to‐noise ratios of *Z*‐PMI and *E*‐PMI lines cannot be quantitatively compared, thus preventing the derivation of the relative populations. We can qualitatively comment that, during the recording of the spectra, we noticed that the intensities of both isomers were comparable. This is probably due to a cooperation of population distribution and differences in the electric dipole moment components.

**Figure 4:**
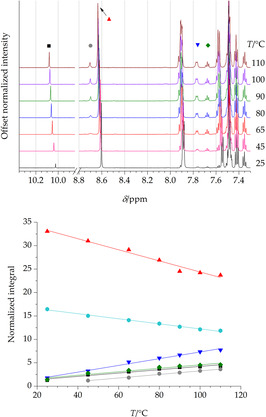
Top panel: NMR signals at different temperatures for HBA and its thermolysis products. Bottom panel: Temperature‐dependence of the normalized integral values (residual solvent peak was fixed to 10) of the NMR signals. In both panels, red triangles refer to HBA CHN (8.63–8.60 ppm), cyan hexagons to aminal HBA (6.06–5.99 ppm), blue upside down triangles to PMI *ortho*‐protons (7.77–7.76 ppm), green diamonds to BA *para*‐proton (7.68 ppm), black squares to BA CHO (10.08–10.02 ppm) and grey circles to PMI CHN (8.70 ppm). The aminal HBA signal is not shown in the top panel for the sake of clarity.

As to the NMR characterization, according to the available literature data, PMI shows two distinct sets (each with two doublets) of signals for the imino hydrogen, with different coupling constants (*J*=16 Hz for the *E* isomer, *J*=25 Hz for the *Z* one).^[26]^ In our analysis, the sample was heated to the desired temperature and the ^1^H NMR spectrum was acquired after 10 minutes to avoid thermalization processes during the acquisition (a complete account of the experiment is provided in the Supporting Information). The formation of both BA and *E*‐PMI was observed as a consequence of the step‐by‐step temperature ramp from 25 °C to 110 °C (see Figure 4). However, no NMR spectroscopic clues denoting the possible formation of BN or *Z*‐PMI were observed, even when the experiments were performed at higher temperature. As far as *Z*‐PMI is concerned, however, our experimental resolution did not allow for unequivocally excluding its presence. This suggests that both experiments (RER and NMR) are characterized by either different hydrolytic mechanisms or the availability of additional pathways in the case of gas‐phase measurements. To confirm the absence of BN in solution after thermal treatment of HBA, a trace amount of the former was added to the NMR samples previously heated at 110 °C and the spectrum was again recorded. The analysis revealed the presence of new signals (doublet at 7.68 ppm, overlapping with a BA triplet; doublet at 7.63 ppm; triplet at 7.50 ppm, overlapping with a HBA multiplet), due to the presence of BN and previously absent. Hence, no BN is formed by hydrolysis of HBA in solution (see Supporting Information). This might suggest that its generation occurs through pathways that rely on metallic catalytic surfaces, such as fast dehydrogenation of PMI.[^43^, ^44^] Indeed, it has been proved that the metal catalysis can take place in the nozzle head in the RER experiment,^[45]^ while this is not the case within the NMR tube.

Figure 4 shows the changes—upon heating—of the diagnostic NMR signals (top) and the temperature‐dependence of their normalized integral values with the respective linear regressions (bottom). Such analysis was performed after an arbitrary assignment to the integral value of the residual peak of 1,1,2,2‐tetrachloroethane‐D_2_ (fixed to 10). The negative‐slope traces correspond to the starting material, that is, HBA, while the positive‐slope traces are attributable to forming species. As expected, the signal relative to the two CHN protons of HBA (red trace) decreases twice as fast as the single‐proton aminal signal (cyan trace).

Two signals (i.e. green and black traces) have almost superimposed trends in terms of both slope and normalized integral values, thus suggesting that the respective protons belong to the same species. Indeed, they are compatible with the CHO and *para*‐proton (triplet) of BA, respectively, thus featuring a 1:1 integration relationship. The other two signals (i.e. blue and gray traces) feature a 2:1 integration relationship. In particular, the singlet at 8.70 ppm (gray trace) is compatible with an iminic CHN proton, while the doublet at 7.77 ppm (blue trace) is compatible with the iminic *ortho*‐protons. Furthermore, the grey trace has a slope which is similar to those observed for the aldehydic signals, thus suggesting that the generation processes of the two species are somehow interconnected. The fact that BA appears to be produced before PMI suggests that the involved hydrolytic mechanism proceeds with the initial formation of BA, followed by the formation of PMI starting from other transient species.

**Scheme 1:**
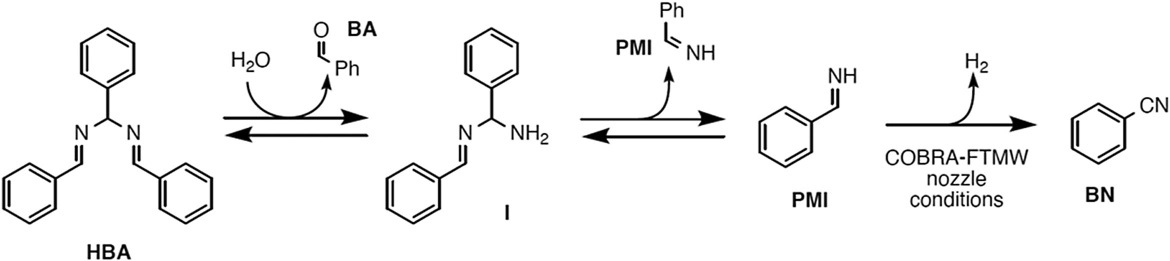
Proposed mechanism for HBA hydrolysis.

Here, we propose a putative hydrolytic pathway (Scheme 1) which is compatible with the aforementioned observations and with previously proposed mechanisms.[^46^, ^47^, ^48^] As noted above, the additional pathway leading to the formation of BN starting from PMI during the microwave spectroscopic measurements is supposed to be a dehydrogenation. In our opinion, the following key points need to be analyzed to explain the proposed mechanism: (i) the role of water; (ii) the role of the metallic surface in the COBRA‐FTMW spectrometer with respect to the glass surfaces acting in the FM‐mmW experiment. Within the former context, water is expected to play a crucial role: the hydrolytic pathway is supposed to proceed as long as water is available. In fact, the amount of water detected by means of the low‐frequency RER experiment as well as the residual water of the deuterated solvent is believed to be sufficient to observe the formation of the main species arising from HBA hydrolysis, that is, BA at room temperature and PMI at higher temperatures.

On the other hand, the FM‐mmW experiment can provide further clues for a deeper understanding of the hydrolytic mechanism. At first, a tentative generation of PMI in the gas phase has been carried out by thermolysis of HBA.

The solid was placed in a glass tube and heated up to 100 °C, also ensuring a uniform heating along the path to the absorption cell. While heating the sample up, a portion of the spectrum around 85.5 GHz was scanned in the attempt of detecting two strong transitions of *E*‐PMI, as predicted by our low frequency measurements. No signal attributable to PMI (nor BN) was found.

Typically, the whole glass apparatus of the FM‐mmW spectrometer is pumped continuously, thereby removing water, although its residual presence cannot be ruled out. However, no metallic surfaces are available in the instrument, thus leading to the formulation of two hypotheses for the lack of formation of PMI in the FM‐mmW experiment: (i) water is not available in a sufficient amount to hydrolyze HBA; (ii) the metal catalysis is mandatory to obtain PMI from HBA in gas phase.

To verify the reliability of spectral predictions and therefore rule out the possibility of false negatives, we adopted a different production method, that is, FVP. By pyrolysis of two possible precursors of PMI (for a vacuum dynamic preparation of PMI, see ref. [49]), that is, benzylamine and *α*‐methyl benzylamine, through dehydrogenation or elimination of CH_4_, respectively, a small set of 27 transitions (belonging only to *E*‐PMI) could be measured, with the latter precursor leading to the highest S/N of the spectra. Conversely, the use of N‐methyl benzylamine gave no signals ascribable to the presence of PMI. BN was found as a pyrolysis co‐product, as proven by recording its rotational transitions. While the PMI signal reached its maximum intensity by setting the furnace temperature to 890 °C, the intensity of BN transitions kept increasing up to 1200 °C. This confirms the prevalence of BN at higher temperatures, in agreement with the low‐frequency RER experiment.

Although a thorough analysis of the mechanisms taking place in the FVP process is beyond the scope of this work, the FM‐mmW experiment proved the reliability of the centimeter‐wave RER measurements and their extrapolation at higher frequencies, but it left some unexplored areas concerning PMI formation from HBA in the gas phase if no metallic surfaces are available. As mentioned above, such possibilities could be further explored going beyond simple thermal conditions. Several attempts to identify the *Z* isomer have been carried out, but no signal ascribable to it was found. This might suggest that only the *E* isomer is generated by ash vacuum pyrolysis, but we did not investigate further this aspect in the present study.

## Conclusions

An easy and affordable approach based on hydrobenzamide thermolysis is presented, which ensures to obtain phenylmethanimine both in gas‐phase and in solution as confirmed by rotational electric resonance (RER) and ^1^H nuclear magnetic resonance (NMR) spectroscopy experiments, respectively. A detailed structural and energetic description of phenylmethanimine has been carried out by resorting to composite schemes for accurate results. This paved the way for the registration and analysis of the microwave spectrum of both *E*‐ and *Z*‐phenylmethanimine, leading to their first laboratory identification. This work is a prerequisite for the possible radio astronomical detection of these species in the interstellar medium, relying on accurate rotational rest frequencies. First, in view of the strong chemical connection between benzonitrile and phenylmethanimine, an astronomical search in the region Taurus Molecular Cloud (TMC‐1) is suggested. Finally, owing to the thorough analysis of RER and NMR spectra at different temperatures, a possible mechanism of phenylmethanimine formation by thermal tuning of hydrobenzamide, in which water is thought to play a crucial role, is also proposed.

## Conflict of interest

The authors declare no conflict of interest.

## Supporting information

As a service to our authors and readers, this journal provides supporting information supplied by the authors. Such materials are peer reviewed and may be re‐organized for online delivery, but are not copy‐edited or typeset. Technical support issues arising from supporting information (other than missing files) should be addressed to the authors.

Supplementary
