# Supplementary material for: A Journey from Thermally Tunable Synthesis to Spectroscopy of Phenylmethanimine in Gas Phase and Solution
Source: Chemistry. 2020 Oct 15;26(65):15016–22. doi: 10.1002/chem.202003270 (PMC7756512; doi:10.1002/chem.202003270)
Supplement: Supplementary file 1 — Supplementary [file CHEM-26-15016-s001.pdf]

Chemistry–A European Journal

Supporting Information

A Journey from Thermally Tunable Synthesis to Spectroscopy of Phenylmethanimine in Gas Phase and Solution

Alessio Melli,[a, b] Simone Potenti,[a, b] Mattia Melosso,[b] Sven Herbers,[c] Lorenzo Spada,*[a, b] Andrea Gualandi,[b] Kevin G. Lengsfeld,[c] Luca Dore,[b] Philipp Buschmann,[c] Pier Giorgio Cozzi,*[b] Jens-Uwe Grabow,*[c] Vincenzo Barone,*[a] and Cristina Puzzarini*[b]

Contents

1 Computational methodology 2

2 RER experiment in gas phase 4

3 NMR experiment in solution 5

4 Synthesis of the precursor 5

5 NMR, IR and MS characterization of HBA 6

6 Rotational transitions of PMI 12

7 QC optimized stationary points of PMI 18

1 Computational methodology

To plan, guide and support rotational electric resonance (RER) spectroscopy experiments, accurate spectral predictions are essential and rely on high-level quantum-chemical (QC) calculations. Indeed, the use of accurate computational strategies provide a mandatory help in the assignment procedure and, often, in the integration of the spectroscopic data.

A preliminary scan of the potential energy surface (PES) of phenylmethanimine (PMI) has been carried out using a cost-effective approach based on density functional theory (DFT). In detail, the B3LYP-D3(BJ)/SNSD level of theory [1,2] (hereafter referred to as B3 level) has been employed for a reliable and fast exploration of the conformational space. The formulation of this hybrid functional includes the treatment of dispersion effects by means of Grimme's DFT-D3 scheme [3] in conjunction with the Becke-Johnson (BJ) damping function. [4] SNSD is double-ζ basis set (available for download at `smart.sns.it`) derived from the N07D one. [5,6] Subsequently, the double-hybrid B2PLYP-D3(BJ) [7] functional has been employed in conjunction with the maug-cc-pVTZ-dH basis set [8] (hereafter altogether denoted as B2 level), to obtain a more accurate description of the stationary points of the PES. In the basis set above, the "-dH" notation refers to the removal of the d functions on hydrogen atoms from the original maug-cc-pVTZ basis. [9] As mentioned in the main text, this investigation of the PES led to the identification of two isomers: E- and Z-PMI.

To allow a straightforward interpretation of their rotational spectrum, the accurate determination of several spectroscopic parameters is mandatory. In this context, the vibrational ground-state rotational constants B_0^i's are defined (where i refers to the a, b, c inertial axes) according to vibrational perturbation theory to the second order (VPT2): [10]

$$B_0^i = B_e^i - \frac{1}{2}\sum_r \alpha_r^i \,, \tag{1}$$

where B_e^i's denoted the equilibrium rotational constants and α_r's are the vibration-rotation interaction constants, the sum running over all r vibrational modes. The B_e^i's are straightforwardly derived from the equilibrium structure, [11] while the second term on the right-hand side is the vibrational correction ($\Delta B_{\text{vib}} = -\frac{1}{2}\sum_r \alpha_r^i$). If we compare the two terms, B_e^i is significantly larger than ΔB_{vib}. [12,13] Thus, the major computational effort is devoted to the equilibrium geometry optimization. For this purpose, we resorted to the "cheap" composite scheme [14] (from here on shortly denoted as ChS). Despite its denomination, which is explained by the limited computational cost compared to a full coupled-cluster (CC) approach, the "cheap" composite scheme leads to accurate results (about 0.001-0.002 Å for bonds and 0.1-0.2° for angles). [14] To fulfil such an accuracy without steeply raising the computational requirements, composite schemes like the ChS approach rely on the additivity approximation, [14] which states that all contributions required can be evaluated at the best possible level of theory according to the size of the system and combined together. Within this

framework, ChS is summarized as follows[14]

$$r_{\text{ChS}} = r(\text{CCSD(T)/VTZ}) + \Delta r(\text{MP2/CBS}) + \Delta r(\text{MP2/CV}) + \Delta r(\text{MP2/AUG})\,, \quad (2)$$

where r denotes a generic structural parameter. On the right-hand side: (i) the first term is r optimized at the fc-CCSD(T)/cc-pVTZ[15,16] level of theory, with "fc-" referring to the frozen-core approximation – and CCSD(T) standing for the CC theory including single and double excitations augmented by a perturbative treatment of triples; (ii) the second term refers to the extrapolation to the complete basis set (CBS) limit, which is carried out using the n^{-3} formula by Helgaker *et al.*[17] applied to Møller-Plesset perturbation theory to second order[18] (MP2), thereby employing fc-MP2/cc-pVTZ and fc-MP2/cc-pVQZ optimized parameters; (iii) the third term is the core-valence (CV) correlation contribution is evaluated as difference between the all-MP2/cc-pCVTZ[19] and fc-MP2/cc-pCVTZ parameters, where "all-" denotes the correlation of all electrons; (iv) the last term introduces the effects of the inclusion of diffuse functions into the basis set, and it is estimated as difference between the fc-MP2/aug-cc-pVTZ[16,20] and fc-MP2/cc-pVTZ optimized parameters.

Subsequently, in order to evaluate the vibrational ground-state rotational constants, as expressed in equation (1), it is necessary to compute the ΔB_{vib} term, which requires expensive anharmonic force-field calculations. However, as anticipated above, the vibrational correction term is significantly smaller – usually 1-3% of the B_0^α value,[12,13] thus allowing us to employ a low, cost-effective level of theory. Therefore, anharmonic force fields (one for each isomer) have been computed at the B3 level.

To complete the set of spectroscopic parameters, the (quartic) centrifugal-distortion constants have been obtained from B2 harmonic force-field calculations. At the same level of theory, first-order properties, such as dipole moment and nuclear quadrupole coupling constants, have also been evaluated. While dipole moments are required to have information on the intensity of rotational transitions, the nuclear quadrupole coupling constants have a strong impact on the rotational spectrum, as explained in the next section.

By reformulating equation (2) as follows, the ChS approach can be employed to derive accurate electronic energy of the stationary points on the investigated PES:

$$E_{\text{ChS}} = E(\text{CCSD(T)/VTZ}) + \Delta E(\text{MP2/CBS}) + \Delta E(\text{MP2/CV})\,. \quad (3)$$

For these single-point energy evaluations, the optimized structures at the B2 level have been chosen as reference geometries. Analogously to equation (2), the first term on the right-hand side of equation (3) is the electronic energy evaluated at the fc-CCSD(T)/cc-pVTZ level of theory, while the last term is derived as difference between all-MP2/cc-pCVTZ and fc-MP2/cc-pCVTZ energies. Different is instead the evaluation of the CBS limit term, which is calculated in two steps: (i) the HF energy is extrapolated to the CBS limit by resorting to the three-point formula proposed by Feller[21] and employing the cc-pVTZ, cc-pVQZ and cc-pV5Z basis sets; (ii) the extrapolation to the

CBS limit of the MP2 correlation energy is derived according to the formula by Helgaker *et al.*,[17] using the cc-pVTZ and the cc-pVQZ basis sets. To incorporate zero-point energy (ZPE) corrections, Chs energies have been augmented by harmonic ZPE terms obtained from the B2 harmonic force-field calculations mentioned above.

Quantum-chemical calculations have been performed using either the `Gaussian16` suite[22] (DFT and MP2) or the `CFOUR` package (coupled cluster).[23]

2 RER experiment in gas phase

The gas-phase characterization of PMI has been performed in the 3-26 GHz range, using the COBRA-type (Coaxially Aligned Beam Resonator Arrangement) Fourier Transform Microwave Spectrometer (FTMW), described in details elsewhere.[24] In this work, the heated head of a nozzle valve filled with hydrobenzamide (HBA) was used to bring the solid sample in the gas-phase. The vapors of thermally-decomposed HBA mixed with neon at backing pressure of 1 bar have been adiabatically expanded into the cavity of the spectrometer through a solenoid valve, resulting in a pulsed supersonic jet. In the spectra, each rotational transition appears as a doublet due to the Doppler effect resulting from the coaxial arrangement between resonator and supersonic jet. Therefore, the arithmetic mean of the Doppler components provides the rest frequency value, whose uncertainty is estimated to be 2 kHz. In agreement with the computed dipole moment components (see Table 1 of the manuscript), both a- and b-type rotational transitions have been observed and assigned for both E and Z isomers of PMI, although only a few b-type components have been fitted for Z-PMI, in accordance with its very low predicted dipole moment along the b axis.

The presence of the ^{14}N atom, which is a quadrupolar nucleus (with nuclear spin is $I = 1$), determines a characteristic pattern of the rotational spectrum, the so-called hyperfine structure. This is due to the nuclear quadrupole coupling (NQC): an interaction between the quadrupole moment and the electric-field gradient at the corresponding nucleus.[11] This interaction is expressed in terms of the nuclear quadrupole-coupling tensor elements χ_{ij}. Owing to the NQC, rotational levels are split in sublevels, according to the $F = J + I$ coupling scheme, with $F = |J + I|, |J + I - 1|, \ldots, |J - I|$. The resulting hyperfine structure has been resolved in most of the recorded transitions in the 3-26 GHz range

Additional spectra were recorded in the 83-100 GHz range using a frequency-modulation millimeter-wave (FM-mmW) spectrometer, described in details elsewhere.[25] In contrast to the previous centimeter-wave measurements, the hyperfine structure was not resolved. In this experiment, a different production method has been employed to generate PMI inside the spectrometer. The vapors of a sample of α-methylbenzylamine were flowed through a flash vacuum pyrolysis (FVP) apparatus,[26,27] constituted by a quartz tube heated at 890 °C by a 30 cm long furnace and connected to the gas inlet of the free-space glass

absorption cell of the spectrometer. The pressure inside the cell was kept at the value of 13 mTorr by continuously pumping out the pyrolysis products. The estimated uncertainity on the line frequency is 10 kHz.

All the transitions of both species have been fitted using the Pickett's CALPGM suite of programs [28] within the I^r representation of Watson's S-reduced Hamiltonian.

3 NMR experiment in solution

A Varian Mercury 400 MHz spectrometer was used to record NMR spectra of HBA, to confirm both its identity and purity. Once HBA was obtained with the required level of purity, a sample was placed in an NMR tube and dissolved in 1,1,2,2-tetrachloroethane-D_2 (b.p. 146 °C) for variable temperature analysis, using a Varian Inova 600 MHz equipped with an ATB Broadband Probe, operating at a field of 14.4 T. The NMR spectra were acquired ten minutes after the chosen temperature was reached, to avoid thermalization processes to occur during the acquisition. Chemical shifts are reported in ppm from TMS with the residual solvent resonance as the internal standard ($CHCl_3$: $\delta = 7.27$ ppm, $C_2H_2Cl_4$: $\delta = 6.0$ ppm).

4 Synthesis of the precursor

HBA was synthesized following the procedure described in literature. [29–32] Ammonium hydroxide solution (28-30% NH_3 basis, 510 mmol, 33.2 mL) was added to benzaldehyde (BA, 39.4 mmol, 4 mL) and the mixture was stirred at room temperature for three hours. A white precipitate appeared, which was recovered by filtration through fritted funnel and washed with small portions of cold diethyl ether (3x10 mL). HBA was obtained as white solid (2.747 g, 9.2 mmol, 70%) and used in the spectroscopic experiments without any further purification (purity >99% by ^{1}H-NMR).
^{1}H-NMR ($CDCl_3$, 400 MHz, ppm) δ: 8.60 (2H, s, *PhCH=N*), 7.90–7.84 (4H, m, *ArH*), 7.55–7.50 (2H, m, *ArH*), 7.47–7.41 (6H, m, *ArH*), 7.41–7.35 (2H, m, *ArH*), 7.33–7.27 (1H, m, *ArH*), 5.99 (1H, s, *CHN$_2$*). ^{13}C-NMR ($CDCl_3$, 100 MHz, ppm) δ: 160.8 (2C), 141.9, 136.2 (2C), 131.2 (2C), 128.8 (4C), 128.70 (4C), 128.68 (2C), 128.0, 127.4 (2C), 92.8. ATR-FTIR (cm^{-1}) $\tilde{\nu}$: 3059, 3031, 2856, 1638, 1492, 1450, 1578, 1357, 1314, 1296, 1216, 1168, 1075, 1030, 914, 755, 700, 632, 565, 483. MS (EI) m/z: 194, 165, 152, 116, 104, 89, 77.

5 NMR, IR and MS characterization of HBA

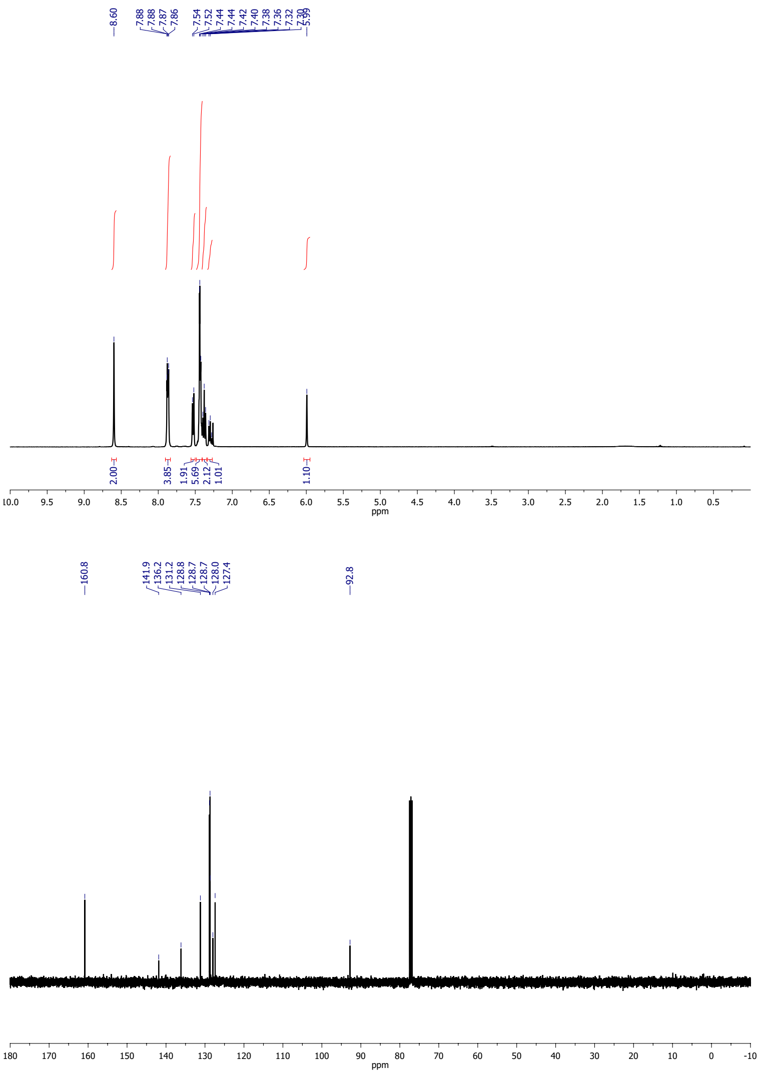

Figure 1: ^{1}H-NMR (400 MHz, $CDCl_3$, 25 °C, top) and ^{13}C-NMR (100 MHz, $CDCl_3$, 25 °C, bottom) of HBA.

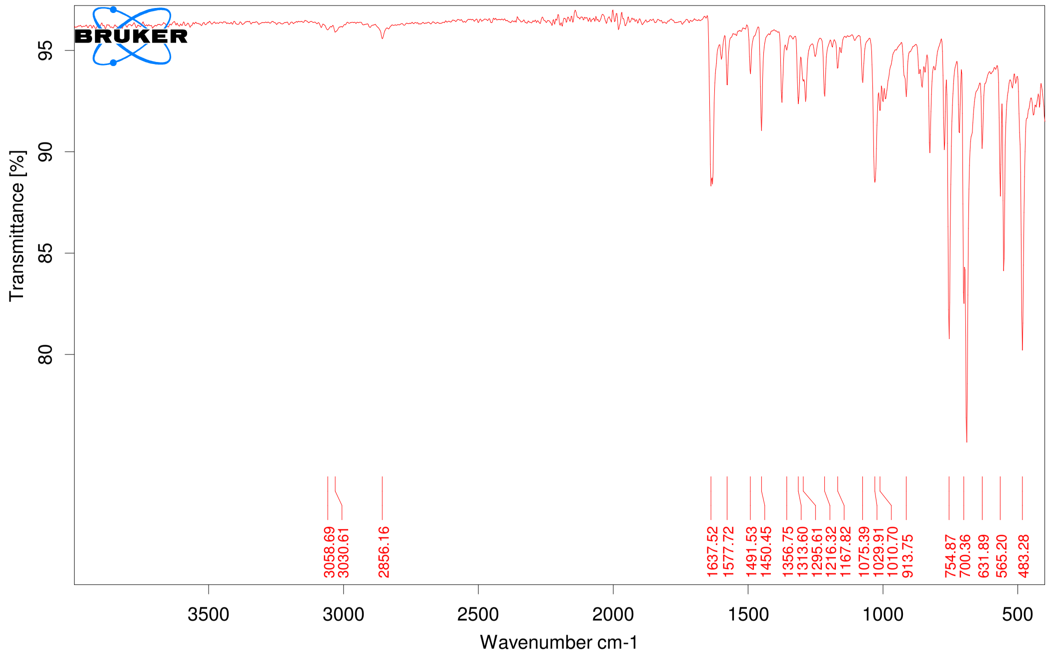

Figure 2: ATR-FTIR of HBA. The spectrum was obtained with an ATR-FTIR Bruker Alpha System spectrometer.

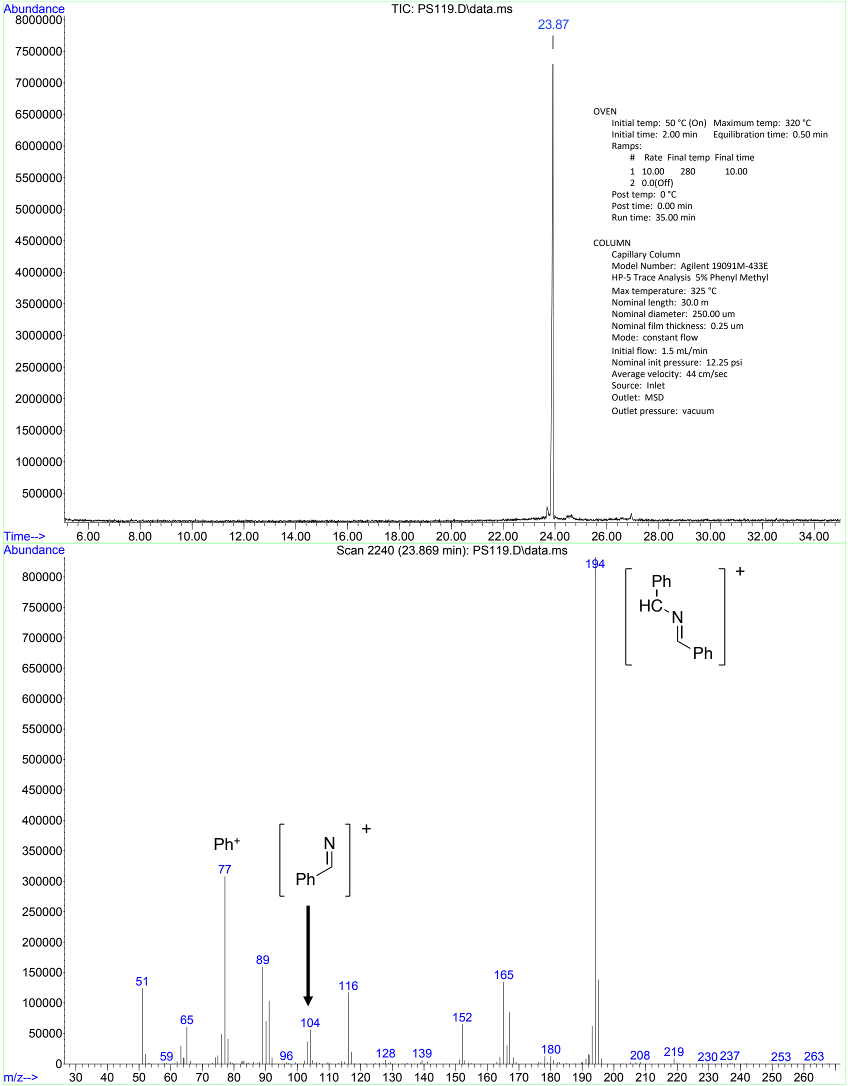

Figure 3: GC-MS analysis of HBA. The spectrum was taken by EI ionization at 70 eV on a Hewlett-Packard 5971 with GC injection.

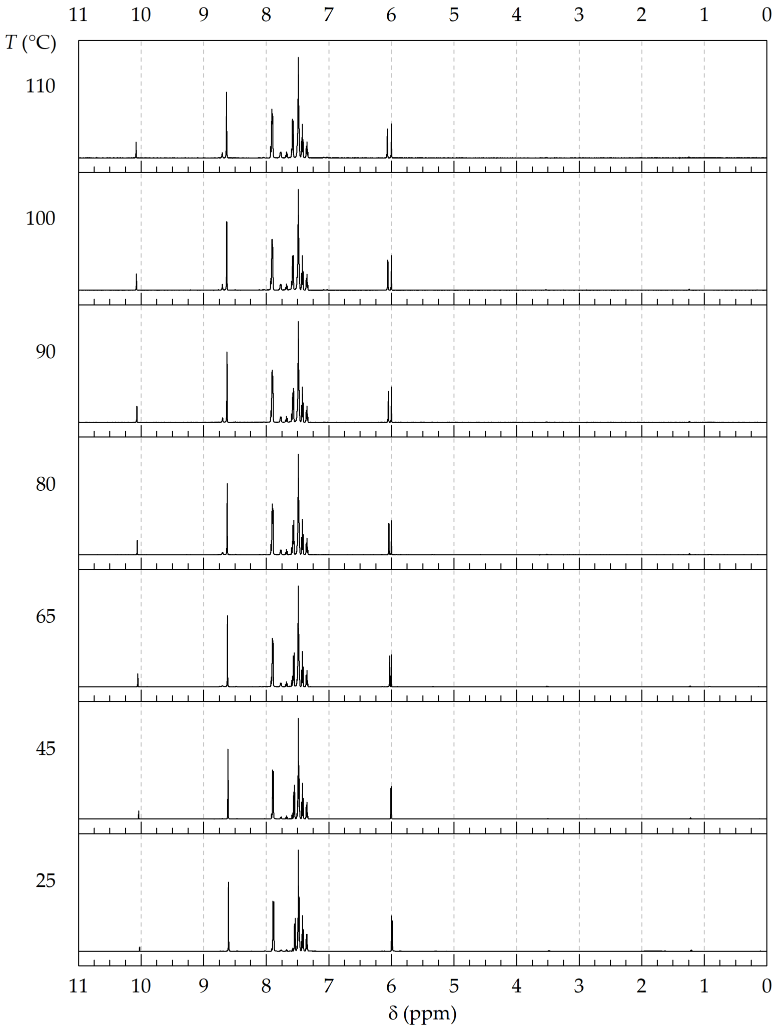

Figure 4: ^{1}H-NMR spectrum (600 MHz, $C_2D_2Cl_4$) of HBA at different temperatures. The reported intensity has been overall normalized with respect to the residual solvent resonance.

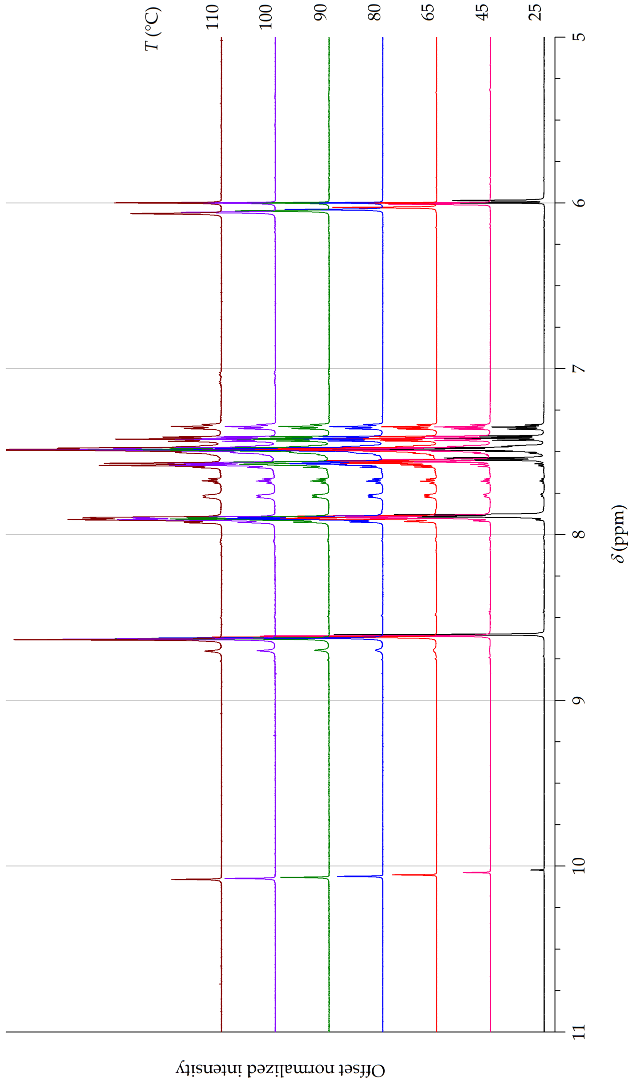

Figure 5: 5-11 ppm range magnification of Figure 4.

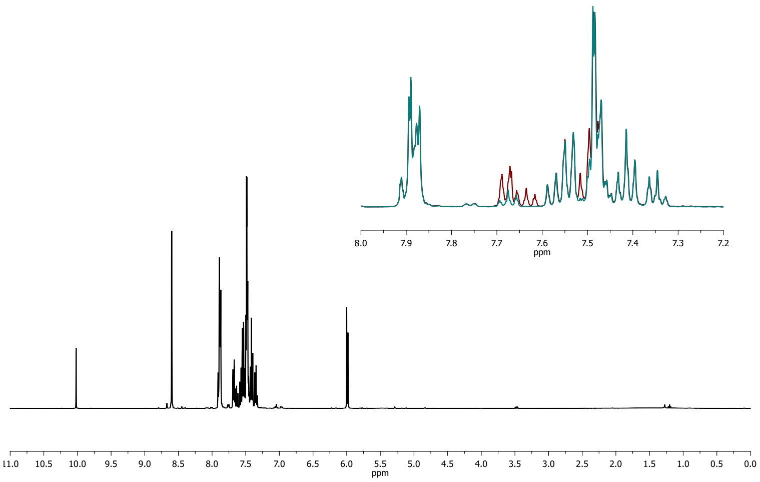

Figure 6: ^{1}H-NMR spectrum (400 MHz, $C_2D_2Cl_4$, 25 °C) of HBA after the thermal gradient with the addition of BN. In the zoomed section, the teal spectrum is before BN addition, while the red one is after it.

6 Rotational transitions of PMI

Table 1: Summary of all the transitions measured in this work for *E*-phenylmethanimine; ν is the frequency in MHz, oc is the difference between the observed frequency and that calculated from the fit procedure expressed in MHz, err is the experimental error assigned to each transition and wt is the weight for blended transitions.

J	K_a	K_c	F	J'	K'_a	K'_c	F'	ν	oc	err	Blended tr.	
											oc	wt
3	0	3	3	2	0	2	2	8208.47460	-0.00033	0.002		
3	0	3	4	2	0	2	3	8208.62501	0.00008	0.002		
6	1	5	5	6	0	6	5	8814.57983	-0.00095	0.002		
2	1	2	3	1	0	1	2	8831.16906	0.00082	0.002		
2	1	2	1	1	0	1	1	8831.38198	0.00022	0.002		
2	1	2	1	1	0	1	0	8832.14251	-0.00265	0.002		
5	2	3	5	5	1	4	5	9756.47810	-0.00086	0.002		
5	2	3	6	5	1	4	6	9756.94268	0.00028	0.002		
5	2	3	4	5	1	4	4	9757.03603	-0.00081	0.002		
4	2	2	4	4	1	3	4	10117.13808	-0.00065	0.002		
4	2	2	5	4	1	3	5	10117.91330	-0.00012	0.002		
4	2	2	3	4	1	3	3	10118.11346	0.00079	0.002		
3	2	1	2	3	1	2	3	10556.50331	-0.00256	0.002		
3	2	1	4	3	1	2	3	10556.60010	0.00123	0.002		
3	2	1	3	3	1	2	3	10556.86522	0.00015	0.002		
3	2	1	4	3	1	2	4	10557.95475	-0.00047	0.002		
3	2	1	3	3	1	2	4	10558.21914	-0.00227	0.002		
4	0	4	4	3	0	3	3	10832.14611	-0.00023	0.002		
4	0	4	5	3	0	3	4	10832.34867	0.00049	0.002		
4	0	4	3	3	0	3	2	10832.40589	-0.00095	0.002		
4	1	4	3	3	1	3	2	10310.09461	-0.00173	0.002		
4	1	4	4	3	1	3	3	10310.13932	-0.00058	0.002		
4	1	4	5	3	1	3	4	10310.17261	-0.00029	0.002		
2	2	0	2	2	1	1	2	10980.28509	0.00225	0.002		
2	2	0	1	2	1	1	1	10982.59320	0.00239	0.002		
5	0	5	4	4	1	4	3	11035.49883	0.00022	0.002		
5	0	5	6	4	1	4	5	11035.67009	0.00070	0.002		
5	0	5	5	4	1	4	4	11036.25693	0.00000	0.002		
3	1	3	3	2	0	2	2	11068.81445	0.00060	0.002		
3	1	3	4	2	0	2	3	11069.95241	0.00037	0.002		
3	1	3	2	2	0	2	1	11070.39513	-0.00142	0.002		
4	1	3	5	3	1	2	4	11746.37019	-0.00067	0.002		
4	1	3	4	3	1	2	3	11746.37903	0.00120	0.002		
4	1	3	3	3	1	2	2	11746.49457	-0.00043	0.002		
2	2	1	1	2	1	2	1	12037.76978	0.00124	0.002		
2	2	1	2	2	1	2	2	12038.71352	0.00197	0.002		
3	2	2	2	3	1	3	2	12594.17207	0.00352	0.002		
3	2	2	4	3	1	3	4	12594.48676	-0.00223	0.002		
3	2	2	3	3	1	3	3	12595.40462	0.00048	0.002		
5	1	5	5	4	1	4	4	12847.13242	0.00018	0.002		
5	1	5	4	4	1	4	3	12847.14561	-0.00032	0.002		
5	1	5	6	4	1	4	5	12847.18567	-0.00006	0.002		
4	1	4	4	3	0	3	3	13170.47871	-0.00011	0.002		
4	1	4	5	3	0	3	4	13171.50011	0.00010	0.002		
4	1	4	3	3	0	3	2	13171.76900	-0.00063	0.002		
4	2	3	3	4	1	4	3	13343.48943	-0.00030	0.002		
4	2	3	5	4	1	4	5	13343.76380	0.00100	0.002		
4	2	3	4	4	1	4	4	13344.82355	-0.00047	0.002		
5	0	5	5	4	0	4	4	13374.58934	-0.00007	0.002		
5	0	5	6	4	0	4	5	13374.82185	0.00064	0.002		
5	0	5	4	4	0	4	3	13374.86136	-0.00004	0.002		
6	0	6	5	5	1	5	4	14029.75034	0.00188	0.002		
6	0	6	7	5	1	5	6	14029.85544	0.00086	0.002		
6	0	6	6	5	1	5	5	14030.25969	0.00005	0.002		
5	2	3	4	4	2	2	3	14266.50912	0.00010	0.002		

(continued)

(continued)

J	K_a	K_c	F	J'	K'_a	K'_c	F'	ν	oc	err	Blended tr. oc	wt
5	2	3	6	4	2	2	5	14266.53178	-0.00035	0.002		
5	2	3	5	4	2	2	4	14266.80065	0.00080	0.002		
5	2	4	4	5	1	5	4	14288.93959	-0.00313	0.002		
5	2	4	6	5	1	5	6	14289.17921	0.00155	0.002		
5	2	4	5	5	1	5	5	14290.32303	-0.00141	0.002		
5	1	4	5	4	1	3	4	14627.45979	0.00017	0.002		
5	1	4	6	4	1	3	5	14627.50370	0.00055	0.002		
5	1	4	4	4	1	3	3	14627.58578	0.00093	0.002		
5	1	5	5	4	0	4	4	15185.46442	-0.00030	0.002		
5	1	5	6	4	0	4	5	15186.33739	-0.00017	0.002		
5	1	5	4	4	0	4	3	15186.50804	-0.00068	0.002		
6	1	6	6	5	1	5	5	15362.48266	-0.00041	0.002		
6	1	6	5	5	1	5	4	15362.52126	-0.00101	0.002		
6	1	6	7	5	1	5	6	15362.54557	-0.00094	0.002		
6	2	5	5	6	1	6	5	15431.30137	-0.00152	0.002		
6	2	5	7	6	1	6	7	15431.50567	0.00063	0.002		
6	2	5	6	6	1	6	6	15432.70159	-0.00198	0.002		
6	0	6	6	5	0	5	5	15841.13522	0.00027	0.002		
6	0	6	7	5	0	5	6	15841.37127	0.00035	0.002		
6	0	6	5	5	0	5	4	15841.39429	-0.00150	0.002		
2	2	1	2	1	1	0	1	16855.41919	-0.00029	0.002		
2	2	1	3	1	1	0	2	16856.61910	0.00163	0.002		
2	2	1	1	1	1	0	0	16858.10620	0.00027	0.002		
2	2	1	1	1	1	0	1	16855.92455	-0.00401	0.002		
2	2	1	2	1	1	0	2	16856.29159	0.00126	0.002		
7	0	7	6	6	1	6	5	16919.52282	-0.00002	0.002		
7	0	7	8	6	1	6	7	16919.58584	0.00023	0.002		
7	0	7	7	6	1	6	6	16919.84231	0.00034	0.002		
6	1	6	6	5	0	5	5	17173.35854	0.00016	0.002		
6	1	6	7	5	0	5	6	17174.06298	0.00012	0.002		
6	1	6	5	5	0	5	4	17174.16984	0.00025	0.002		
2	2	0	1	1	1	1	0	17240.78193	0.00097	0.002		
2	2	0	2	1	1	1	2	17242.20025	0.00184	0.002		
2	2	0	3	1	1	1	2	17242.42158	0.00003	0.002		
2	2	0	2	1	1	1	1	17243.37434	-0.00052	0.002		
2	2	0	1	1	1	1	1	17243.72362	0.00140	0.002		
6	1	5	6	5	1	4	5	17465.37842	-0.00054	0.002		
6	1	5	7	5	1	4	6	17465.46274	-0.00004	0.002		
6	1	5	5	5	1	4	4	17465.52250	-0.00045	0.002		
7	1	7	7	6	1	6	6	17856.42050	0.00004	0.002		
7	1	7	6	6	1	6	5	17856.47110	-0.00064	0.002		
7	1	7	8	6	1	6	7	17856.48829	0.00005	0.002		
6	3	3	6	6	2	4	6	18070.56579	-0.00009	0.002		
6	3	3	7	6	2	4	7	18071.29051	0.00269	0.002		
7	0	7	7	6	0	6	6	18252.06506	-0.00034	0.002		
7	0	7	8	6	0	6	7	18252.27818	0.00063	0.002		
7	0	7	6	6	0	6	5	18252.29594	-0.00070	0.002		
5	3	2	5	5	2	3	5	18560.77530	-0.00050	0.002		
5	3	2	6	5	2	3	6	18561.44224	0.00293	0.002		
5	3	2	4	5	2	3	4	18561.57336	-0.00111	0.002		
4	3	1	4	4	2	2	4	18878.79790	-0.00103	0.002		
4	3	1	5	4	2	2	5	18879.41582	0.00190	0.002		
4	3	1	3	4	2	2	3	18879.57320	0.00114	0.002		
3	3	0	3	3	2	1	3	19051.54080	-0.00207	0.002		
3	3	0	4	3	2	1	4	19052.19468	-0.00288	0.002		
3	3	1	3	3	2	2	3	19176.90962	-0.00337	0.002		
3	3	1	4	3	2	2	4	19177.30131	-0.00388	0.002		
7	1	7	8	6	0	6	7	19189.17967	-0.00051	0.002		
7	1	7	6	6	0	6	5	19189.24545	-0.00010	0.002		
4	3	2	4	4	2	3	4	19244.53320	-0.00085	0.002		
4	3	2	5	4	2	3	5	19244.69083	0.00185	0.002		
4	3	2	3	4	2	3	3	19244.73090	0.00198	0.002		
3	2	2	2	2	1	1	2	19264.49271	-0.00035	0.002	-0.00037	0.5000

(continued)

(continued)

J	K_a	K_c	F	J'	K'_a	K'_c	F'	ν	oc	err	Blended tr.	
											oc	wt
3	2	2	3	2	1	1	2	19264.49271	-0.00039	0.002	-0.00037	0.5000
3	2	2	4	2	1	1	3	19265.75447	0.00104	0.002	0.00099	0.5000
3	2	2	3	2	1	1	3	19265.75447	0.00093	0.002	0.00099	0.5000
3	2	2	2	2	1	1	1	19266.45386	0.00019	0.002		
5	3	3	4	5	2	4	4	19377.30386	0.00310	0.002	0.00093	0.3333
5	3	3	6	5	2	4	6	19377.30386	0.00300	0.002	0.00093	0.3333
5	3	3	5	5	2	4	5	19377.30386	-0.00331	0.002	0.00093	0.3333
6	3	4	5	6	2	5	5	19601.69404	0.00006	0.002		
6	3	4	7	6	2	5	7	19601.71904	0.00127	0.002		
8	0	8	7	7	1	7	6	19696.43450	-0.00034	0.002		
8	0	8	9	7	1	7	8	19696.47394	0.00051	0.002		
8	0	8	8	7	1	7	7	19696.61789	0.00081	0.002		
7	1	6	7	6	1	5	6	20246.04109	-0.00023	0.002		
7	1	6	8	6	1	5	7	20246.16495	0.00204	0.002		
7	1	6	6	6	1	5	5	20246.20976	-0.00106	0.002		
3	2	1	2	2	1	2	1	20473.11405	0.00119	0.002		
3	2	1	4	2	1	2	3	20473.72418	-0.00056	0.002		
3	2	1	3	2	1	2	2	20474.92474	0.00059	0.002		
8	1	8	8	7	0	7	7	21267.21815	-0.00050	0.002		
8	1	8	9	7	0	7	8	21267.61060	-0.00057	0.002		
8	1	8	7	7	0	7	6	21267.64513	-0.00026	0.002		
4	2	3	4	3	1	2	3	21489.92885	0.00016	0.002		
4	2	3	5	3	1	2	4	21491.17305	0.00121	0.002		
4	2	3	3	3	1	2	2	21491.61770	0.00033	0.002		
9	0	9	8	8	1	8	7	22371.31773	0.00146	0.002		
9	0	9	10	8	1	8	9	22371.34116	0.00049	0.002		
9	0	9	9	8	1	8	8	22371.40814	0.00054	0.002		
9	1	9	9	8	0	8	8	23421.86330	-0.00021	0.002		
9	1	9	10	8	0	8	9	23422.14174	-0.00023	0.002		
9	1	9	8	8	0	8	7	23422.15675	-0.00215	0.002		
5	2	4	5	4	1	3	4	23536.18320	-0.00031	0.002		
5	2	4	6	4	1	3	5	23537.40248	0.00091	0.002		
5	2	4	4	4	1	3	3	23537.72138	0.00009	0.002		
4	2	2	3	3	1	3	2	24026.57685	0.00047	0.002		
4	2	2	5	3	1	3	4	24027.04816	0.00002	0.002		
4	2	2	4	3	1	3	3	24028.55167	-0.00012	0.002		
6	2	5	6	5	1	4	5	25413.58610	-0.00000	0.002		
6	2	5	7	5	1	4	6	25414.77261	0.00030	0.002		
6	2	5	5	5	1	4	4	25415.01766	-0.00121	0.002		
34	0	34		33	1	33		83107.633	-0.00156	0.010	-0.00246	0.4080
34	1	34		33	1	33		83107.633	-0.00220	0.010	-0.00246	0.0920
34	0	34		33	0	33		83107.633	-0.00271	0.010	-0.00246	0.0920
34	1	34		33	0	33		83107.633	-0.00335	0.010	-0.00246	0.4080
33	1	32		32	2	31		83111.976	0.11538	0.010	-0.00288	0.3995
33	2	32		32	2	31		83111.976	0.02808	0.010	-0.00288	0.1005
33	1	32		32	1	31		83111.976	-0.03383	0.010	-0.00288	0.1005
33	2	32		32	1	31		83111.976	-0.12113	0.010	-0.00288	0.3995
35	0	35		34	1	34		85516.228	0.00023	0.010	-0.00028	0.4081
35	1	35		34	1	34		85516.228	-0.00014	0.010	-0.00028	0.0919
35	0	35		34	0	34		85516.228	-0.00042	0.010	-0.00028	0.0919
35	1	35		34	0	34		85516.228	-0.00078	0.010	-0.00028	0.4081
34	1	33		33	2	32		85520.102	0.09767	0.010	0.01064	0.3145
34	2	33		33	2	32		85520.102	0.04672	0.010	0.01064	0.0787
34	1	33		33	1	32		85520.102	0.01037	0.010	0.01064	0.0787
34	2	33		33	1	32		85520.102	-0.04058	0.010	0.01064	0.3145
32	3	29		31	4	28		85520.102	-0.05526	0.010	0.01064	0.2137
36	0	36		35	1	35		87924.807	0.00794	0.010	0.00765	0.4082
36	1	36		35	1	35		87924.807	0.00773	0.010	0.00765	0.0918
36	0	36		35	0	35		87924.807	0.00757	0.010	0.00765	0.0918
36	1	36		35	0	35		87924.807	0.00737	0.010	0.00765	0.4082
35	1	34		34	2	33		87928.199	0.04273	0.010	0.00243	0.4003
35	2	34		34	2	33		87928.199	0.01307	0.010	0.00243	0.0997
35	1	34		34	1	33		87928.199	-0.00822	0.010	0.00243	0.0997

(continued)

(continued)

J	K_a	K_c	F	J'	K'_a	K'_c	F'	ν	oc	err	Blended tr. oc	wt
35	2	34		34	1	33		87928.199	-0.03787	0.010	0.00243	0.4003
37	0	37		36	1	36		90333.348	0.00068	0.010	0.00052	0.4083
37	1	37		36	1	36		90333.348	0.00056	0.010	0.00052	0.0917
37	0	37		36	0	36		90333.348	0.00047	0.010	0.00052	0.0917
37	1	37		36	0	36		90333.348	0.00036	0.010	0.00052	0.4083
36	1	35		35	2	34		90336.331	0.01401	0.010	-0.00943	0.4007
36	2	35		35	2	34		90336.331	-0.00321	0.010	-0.00943	0.0993
36	1	35		35	1	34		90336.331	-0.01565	0.010	-0.00943	0.0993
36	2	35		35	1	34		90336.331	-0.03287	0.010	-0.00943	0.4007
34	2	32		33	3	31		87949.735	0.00232	0.010		
34	3	32		33	2	31		87954.494	-0.01042	0.010		
33	3	30		32	4	29		87958.796	0.00065	0.010		
35	2	33		34	3	32		90356.804	0.00204	0.010		
35	3	33		34	3	32		90357.860	-0.03029	0.020		
35	2	33		34	2	32		90358.622	0.01852	0.020		
35	3	33		34	2	32		90359.694	0.00219	0.010		
43	3	40		43	2	41		90362.768	0.00331	0.010		
43	4	40		43	3	41		90363.240	-0.00422	0.010		
34	3	31		33	4	30		90384.351	0.00800	0.010		
34	4	31		33	4	30		90421.744	0.02902	0.020		
38	0	38		37	1	37		92741.868	-0.00347	0.010	-0.00356	0.4084
38	0	38		37	0	37		92741.868	-0.00358	0.010	-0.00356	0.0916
38	1	38		37	1	37		92741.868	-0.00353	0.010	-0.00356	0.0916
38	1	38		37	0	37		92741.868	-0.00365	0.010	-0.00356	0.4084
37	1	36		36	2	35		92744.492	0.00724	0.010	-0.00635	0.4010
37	2	36		36	2	35		92744.492	-0.00273	0.010	-0.00635	0.0990
37	1	36		36	1	35		92744.492	-0.00998	0.010	-0.00635	0.0990
37	2	36		36	1	35		92744.492	-0.01995	0.010	-0.00635	0.4010
36	2	34		35	3	33		92763.756	0.01620	0.010		
36	3	34		35	2	33		92765.460	-0.02318	0.010		
39	0	39		38	1	38		95150.367	-0.00346	0.010	-0.00351	0.4085
39	1	39		38	1	38		95150.367	-0.00350	0.010	-0.00351	0.0915
39	0	39		38	0	38		95150.367	-0.00353	0.010	-0.00351	0.0915
39	1	39		38	0	38		95150.367	-0.00356	0.010	-0.00351	0.4085
38	1	37		37	2	36		95152.670	0.01328	0.010	0.00541	0.4013
38	2	37		37	2	36		95152.670	0.00751	0.010	0.00541	0.0987
38	1	37		37	1	36		95152.670	0.00330	0.010	0.00541	0.0987
38	2	37		37	1	36		95152.670	-0.00247	0.010	0.00541	0.4013
40	0	40		39	0	39		97558.847	0.00366	0.010	0.00367	0.0915
40	0	40		39	1	39		97558.847	0.00370	0.010	0.00367	0.4085
40	1	40		39	0	39		97558.847	0.00364	0.010	0.00367	0.4085
40	1	40		39	1	39		97558.847	0.00368	0.010	0.00367	0.0915
39	1	38		38	2	37		97560.833	0.00340	0.010	-0.00115	0.4017
39	2	38		38	2	37		97560.833	0.00007	0.010	-0.00115	0.0983
39	1	38		38	1	37		97560.833	-0.00237	0.010	-0.00115	0.0983
39	2	38		38	1	37		97560.833	-0.00570	0.010	-0.00115	0.4017

Table 2: Summary of all the transitions measured in this work for *Z*-phenylmethanimine; ν is the frequency in MHz, oc is the difference between the observed frequency and that calculated from the fit procedure expressed in MHz, err is the experimental error assigned to each transition and wt is the weight for blended transitions.

J	K_a	K_c	F	J'	K'_a	K'_c	F'	ν	oc	err	Blended tr. oc	wt
2	1	2	2	1	1	1	1	5132.51819	0.00455	0.002		
2	1	2	3	1	1	1	2	5133.75354	-0.00044	0.002		
2	0	2	1	1	0	1	0	5462.13612	0.00086	0.002		
2	0	2	2	1	0	1	1	5463.07668	-0.00012	0.002		
2	0	2	3	1	0	1	2	5463.18572	-0.00056	0.002		

(continued)

(continued)

J	K_a	K_c	F	J'	K'_a	K'_c	F'	ν	oc	err	Blended tr. oc	wt
2	1	1	2	1	1	0	1	5840.76633	-0.00032	0.002		
2	1	1	3	1	1	0	2	5841.96436	-0.00023	0.002		
2	1	1	1	1	1	0	0	5843.46992	0.00248	0.002		
3	1	3	3	2	1	2	2	7685.15636	-0.00053	0.002		
3	1	3	2	2	1	2	1	7685.46409	0.00294	0.002		
3	1	3	4	2	1	2	3	7685.52647	-0.00047	0.002		
3	0	3	2	2	0	2	1	8133.92445	-0.00065	0.002		
3	0	3	3	2	0	2	2	8134.07248	-0.00069	0.002		
3	0	3	4	2	0	2	3	8134.15918	-0.00033	0.002		
3	2	2	3	2	2	1	2	8230.44543	0.00072	0.002		
3	2	2	4	2	2	1	3	8231.70883	0.00279	0.002		
3	2	2	2	2	2	1	1	8232.40842	0.00134	0.002		
3	2	1	3	2	2	0	2	8327.80029	0.00037	0.002		
3	2	1	4	2	2	0	3	8329.02713	0.00548	0.002		
3	2	1	2	2	2	0	1	8329.71703	0.00298	0.002		
3	1	2	3	2	1	1	2	8746.46866	-0.00075	0.002		
3	1	2	4	2	1	1	3	8746.81602	-0.00070	0.002		
3	1	2	2	2	1	1	1	8746.87371	-0.00010	0.002		
4	1	4	4	3	1	3	3	10220.86137	-0.00088	0.002		
4	1	4	3	3	1	3	2	10220.95890	-0.00115	0.002		
4	1	4	5	3	1	3	4	10221.03771	0.00015	0.002		
4	0	4	3	3	0	3	2	10736.70041	-0.00063	0.002		
4	0	4	4	3	0	3	3	10736.72629	-0.00016	0.002		
4	0	4	5	3	0	3	4	10736.80709	-0.00063	0.002		
4	2	3	4	3	2	2	3	10955.73276	-0.00082	0.002		
4	2	3	5	3	2	2	4	10956.27498	-0.00007	0.002		
4	2	3	3	3	2	2	2	10956.41361	-0.00057	0.002		
4	3	2	4	3	3	1	3	11020.36090	0.00147	0.002		
4	3	2	5	3	3	1	4	11021.51653	0.00191	0.002		
4	3	1	5	3	3	0	4	11027.88027	-0.00122	0.002		
4	2	2	4	3	2	1	3	11194.19919	-0.00052	0.002		
4	2	2	5	3	2	1	4	11194.69005	0.00036	0.002		
4	2	2	3	3	2	1	2	11194.82249	0.00088	0.002		
4	1	3	4	3	1	2	3	11630.99244	-0.00051	0.002		
4	1	3	3	3	1	2	2	11631.13641	-0.00007	0.002		
4	1	3	5	3	1	2	4	11631.15519	-0.00073	0.002		
5	1	5	5	4	1	4	4	12736.94786	-0.00064	0.002		
5	1	5	4	4	1	4	3	12736.99484	-0.00002	0.002		
5	1	5	6	4	1	4	5	12737.05281	-0.00059	0.002		
5	0	5	5	4	0	4	4	13260.43347	0.00097	0.002		
5	0	5	4	4	0	4	3	13260.44918	-0.00144	0.002		
5	0	5	6	4	0	4	5	13260.51129	-0.00086	0.002		
5	2	4	5	4	2	3	4	13664.34461	-0.00043	0.002		
5	2	4	6	4	2	3	5	13664.63531	0.00023	0.002		
5	2	4	4	4	2	3	3	13664.66443	-0.00073	0.002		
5	3	3	6	4	3	2	5	13792.84439	-0.00246	0.002		
5	3	3	4	4	3	2	3	13792.99516	0.00108	0.002		
5	3	2	5	4	3	1	4	13814.39515	-0.00012	0.002		
5	3	2	6	4	3	1	5	13814.98514	0.00071	0.002		
5	3	2	4	4	3	1	3	13815.12966	-0.00100	0.002		
5	2	3	5	4	2	2	4	14122.63274	-0.00131	0.002		
5	2	3	6	4	2	2	5	14122.86480	-0.00035	0.002		
5	2	3	4	4	2	2	3	14122.88923	-0.00022	0.002		
5	1	4	5	4	1	3	4	14485.42326	-0.00031	0.002		
5	1	4	4	4	1	3	3	14485.49861	-0.00176	0.002		
5	1	4	6	4	1	3	5	14485.52334	-0.00060	0.002		
6	1	6	6	5	1	5	5	15231.97344	0.00020	0.002		
6	1	6	5	5	1	5	4	15232.00161	-0.00069	0.002		
6	1	6	7	5	1	5	6	15232.04549	-0.00035	0.002		
4	2	3	3	4	0	4	3	15656.35162	0.00067	0.002		
4	2	3	5	4	0	4	5	15656.58741	0.00178	0.002		
4	2	3	4	4	0	4	4	15657.49719	-0.00140	0.002		
6	0	6	6	5	0	5	5	15709.68982	0.00102	0.002		

(continued)

(continued)

J	K_a	K_c	F	J'	K'_a	K'_c	F'	ν	oc	err	Blended tr.	
											oc	wt
6	0	6	5	5	0	5	4	15709.72402	0.00011	0.002		
6	0	6	7	5	0	5	6	15709.76465	0.00002	0.002		
5	2	4	4	5	0	5	4	16060.56469	-0.00080	0.002		
5	2	4	6	5	0	5	6	16060.71097	0.00242	0.002		
5	2	4	5	5	0	5	5	16061.40876	-0.00236	0.002		
6	2	5	6	5	2	4	5	16352.70448	-0.00078	0.002		
6	2	5	7	5	2	4	6	16352.88449	0.00010	0.002	16352.88500	0.5000
6	2	5	5	5	2	4	4	16352.88449	-0.00111	0.002	16352.88500	0.5000
6	3	4	6	5	3	3	5	16569.55257	-0.00105	0.002		
6	3	4	7	5	3	3	6	16569.90078	-0.00009	0.002		
6	3	4	5	5	3	3	4	16569.95520	-0.00052	0.002		
6	3	3	7	5	3	2	6	16628.21450	-0.00347	0.002		
6	3	3	5	5	3	2	4	16628.27373	0.00237	0.002		
2	2	1	3	1	1	0	2	16797.13357	0.00101	0.002		
2	2	1	2	1	1	0	1	16797.50532	-0.00282	0.002		
6	2	4	6	5	2	3	5	17104.46080	-0.00043	0.002		
6	2	4	5	5	2	3	4	17104.58014	0.00301	0.002	17104.57856	0.5000
6	2	4	7	5	2	3	6	17104.58014	0.00014	0.002	17104.57856	0.5000
2	2	0	3	1	1	1	2	17175.68103	0.00138	0.002		
6	1	5	6	5	1	4	5	17298.36856	-0.00012	0.002		
6	1	5	5	5	1	4	4	17298.42432	-0.00046	0.002		
6	1	5	7	5	1	4	6	17298.44422	-0.00002	0.002		
7	1	7	7	6	1	6	6	17706.10175	-0.00090	0.002		
7	1	7	6	6	1	6	5	17706.12452	-0.00017	0.002		
7	1	7	8	6	1	6	7	17706.15781	-0.00007	0.002		
7	0	7	7	6	0	6	6	18103.54576	0.00048	0.002		
7	0	7	6	6	0	6	5	18103.58414	0.00015	0.002		
7	0	7	8	6	0	6	7	18103.61343	-0.00031	0.002		
7	2	6	7	6	2	5	6	19017.34084	0.00090	0.002		
7	2	6	6	6	2	5	5	19017.46340	0.00777	0.002	19017.45921	0.5000
7	2	6	8	6	2	5	7	19017.46340	0.00060	0.002	19017.45921	0.5000
7	3	5	7	6	3	4	6	19349.19936	0.00060	0.002		
7	3	5	8	6	3	4	7	19349.41967	-0.00007	0.002		
7	3	5	6	6	3	4	5	19349.43800	-0.00241	0.002		
7	3	4	7	6	3	3	6	19477.74442	0.00036	0.002		
7	3	4	8	6	3	3	7	19477.95055	0.00230	0.002		
7	3	4	6	6	3	3	5	19477.96467	-0.00231	0.002		
7	1	6	7	6	1	5	6	20056.33672	0.00073	0.002		
7	1	6	6	6	1	5	5	20056.38754	0.00038	0.002		
7	1	6	8	6	1	5	7	20056.40194	-0.00025	0.002		
7	2	5	7	6	2	4	6	20115.84586	-0.00006	0.002		
7	2	5	6	6	2	4	5	20115.90309	-0.00022	0.002		
7	2	5	8	6	2	4	7	20115.91281	0.00005	0.002		
8	1	8	8	7	1	7	7	20160.91119	0.00084	0.002		
8	1	8	7	7	1	7	6	20160.92793	-0.00101	0.002		
8	1	8	9	7	1	7	8	20160.95480	-0.00015	0.002		
8	0	8	8	7	0	7	7	20466.94256	0.00104	0.002		
8	0	8	7	7	0	7	6	20466.97531	-0.00174	0.002		
8	0	8	9	7	0	7	8	20467.00107	0.00070	0.002		
8	2	7	8	7	2	6	7	21655.23989	0.00127	0.002		
8	1	7	8	7	1	6	7	22744.82889	0.00001	0.002		
9	1	8	9	8	1	7	8	25351.20442	0.00154	0.002		
9	1	8	8	8	1	7	7	25351.25816	-0.00140	0.002		

7 QC optimized stationary points of PMI

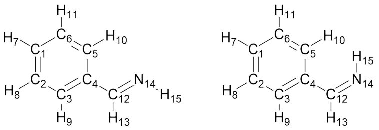

Figure 7: Atom numbering of *E*- (left) and *Z*- (right) phenylmethanimine.

Parameter	*E*-phenylmethanimine		*E*-phenylmethanimine		$TS_{E\text{-}Z}$
	ChS	B2	ChS	B2	B2
r C_1-C_2	1.3895	1.3904	1.3895	1.3903	1.3909
r C_2-C_3	1.3916	1.3909	1.3910	1.3902	1.3911
r C_3-C_4	1.3938	1.3958	1.3948	1.3968	1.3943
r C_4-C_5	1.3975	1.3989	1.3968	1.3982	1.3958
r C_5-C_6	1.3865	1.3863	1.3872	1.3870	1.3869
r C_1-H_7	1.0816	1.0808	1.0813	1.0806	1.0809
r C_2-H_8	1.0814	1.0806	1.0813	1.0805	1.0807
r C_3-H_9	1.0836	1.0824	1.0833	1.0821	1.0829
r C_5-H_{10}	1.0814	1.0800	1.0829	1.0816	1.0812
r C_6-H_{11}	1.0816	1.0808	1.0814	1.0806	1.0810
r C_{12}-C_4	1.4708	1.4699	1.4785	1.4775	1.4886
r C_{12}-H_{13}	1.0936	1.0941	1.0901	1.0902	1.1061
r C_{12}-N_{14}	1.2763	1.2755	1.2748	1.2739	1.2395
r N_{14}-H_{15}	1.0165	1.0173	1.0199	1.0209	0.9876
$\angle$ C_3-C_2-C_1	119.73	119.77	119.79	119.83	119.75
$\angle$ C_4-C_3-C_2	120.42	120.52	120.75	120.83	120.49
$\angle$ C_5-C_4-C_3	119.65	119.41	119.02	118.82	119.46
$\angle$ C_6-C_5-C_4	119.90	120.04	120.36	120.48	120.20
$\angle$ H_7-C_1-C_2	120.00	120.02	120.15	120.15	120.01
$\angle$ H_8-C_2-C_1	120.19	120.18	120.18	120.17	120.11
$\angle$ H_9-C_3-C_2	120.10	120.04	120.06	120.02	120.27
$\angle$ H_{10}-C_5-C_4	118.80	118.66	120.34	120.17	118.57
$\angle$ H_{11}-C_6-C_5	119.90	119.87	119.83	119.83	120.05
$\angle$ C_{12}-C_4-C_3	119.19	119.32	118.21	118.37	119.05
$\angle$ H_{13}-C_{12}-C_4	115.31	114.78	114.64	114.25	112.24
$\angle$ N_{14}-C_{12}-C_4	122.10	122.48	128.64	129.21	126.12
$\angle$ H_{15}-N_{14}-C_{12}	110.77	110.78	111.55	111.39	179.15

[a] No dihedral angles have been reported because all stationary points are planar.
Bonds in Å, angles in degrees.

Table 3: Structural parameters of the *E*- and *Z*-phenylmethanimine isomers and of $TS_{E\text{-}Z}$.

References

[1] A. D. Becke, *Phys. Rev. A* **1988**, *38*, 3098.

[2] C. Lee, W. Yang, R. G. Parr, *Phys. Rev. B* **1988**, *37*, 785.

[3] S. Grimme, J. Antony, S. Ehrlich, H. Krieg, *J. Chem. Phys.* **2010**, *132*, 154104.

[4] S. Grimme, S. Ehrlich, L. Goerigk, *J. Comput. Chem.* **2011**, *32*, 1456–1465.

[5] V. Barone, P. Cimino, *Chem. Phys. Lett.* **2008**, *454*, 139–143.

[6] V. Barone, P. Cimino, E. Stendardo, *J. Chem. Theory Comput.* **2008**, *4*, 751–764.

[7] S. Grimme, *J. Chem. Phys.* **2006**, *124*, 034108.

[8] T. Fornaro, M. Biczysko, J. Bloino, V. Barone, *Phys. Chem. Chem. Phys.* **2016**, *18*, 8479–8490.

[9] E. Papajak, H. R. Leverentz, J. Zheng, D. G. Truhlar, *J. Chem. Theory Comput.* **2009**, *5*, 1197–1202.

[10] I. M. Mills in *3.2 Vibration-Rotation Structure in Asymmetric- and Symmetric-Top Molecules*, *Vol. 1*, Academic Press, **1972**, p. 115.

[11] W. Gordy, R. L. Cook, *Microwave Molecular Spectra*, Wiley, **1984**.

[12] C. Puzzarini, M. Heckert, J. Gauss, *J. Chem. Phys.* **2008**, *128*, 194108.

[13] V. Barone, M. Biczysko, C. Puzzarini, *Accounts Chem. Res.* **2015**, *48*, 1413–1422.

[14] C. Puzzarini, V. Barone, *Phys. Chem. Chem. Phys.* **2011**, *13*, 7189–7197.

[15] K. Raghavachari, G. W. Trucks, J. A. Pople, M. Head-Gordon, *Chem. Phys. Lett.* **1989**, *157*, 479–483.

[16] T. H. Dunning, *J. Chem. Phys.* **1989**, *90*, 1007–1023.

[17] T. Helgaker, W. Klopper, H. Koch, J. Noga, *J. Chem. Phys.* **1997**, *106*, 9639–9646.

[18] C. Møller, M. S. Plesset, *Phys. Rev.* **1934**, *46*, 618.

[19] D. E. Woon, T. H. Dunning Jr, *J. Chem. Phys.* **1995**, *103*, 4572–4585.

[20] R. A. Kendall, T. H. Dunning, R. J. Harrison, *J. Chem. Phys.* **1992**, *96*, 6796–6806.

[21] D. Feller, *J. Chem. Phys.* **1993**, *98*, 7059–7071.

[22] M. J. Frisch, G. W. Trucks, H. B. Schlegel, G. E. Scuseria, M. A. Robb, J. R. Cheeseman, G. Scalmani, V. Barone, G. A. Petersson, H. Nakatsuji, X. Li, M. Caricato, A. V. Marenich, J. Bloino, B. G. Janesko, R. Gomperts, B. Mennucci, H. P. Hratchian, J. V. Ortiz, A. F. Izmaylov, J. L. Sonnenberg, D. Williams-Young, F. Ding, F. Lipparini, F. Egidi, J. Goings, B. Peng, A. Petrone, T. Henderson, D. Ranasinghe, V. G. Zakrzewski, J. Gao, N. Rega, G. Zheng, W. Liang, M. Hada, M. Ehara, K. Toyota, R. Fukuda, J. Hasegawa, M. Ishida, T. Nakajima, Y. Honda, O. Kitao, H. Nakai, T. Vreven, K. Throssell, J. A. Montgomery, Jr., J. E. Peralta, F. Ogliaro, M. J. Bearpark, J. J. Heyd, E. N. Brothers, K. N. Kudin, V. N. Staroverov, T. A. Keith, R. Kobayashi, J. Normand, K. Raghavachari, A. P. Rendell, J. C. Burant, S. S. Iyengar, J. Tomasi, M. Cossi, J. M. Millam, M. Klene, C. Adamo, R. Cammi, J. W. Ochterski, R. L. Martin, K. Morokuma, O. Farkas, J. B. Foresman, D. J. Fox, *Gaussian16*, **2016**, Wallingford CT. See `http://gaussian.com`.

[23] J. F. Stanton, J. Gauss, M. E. Harding, P. G. Szalay, *CFOUR, Coupled-Cluster techiques for Computational Chemistry*, **2008**, See `http://www.cfour.de`.

[24] J.-U. Grabow, W. Stahl, H. Dreizler, *Rev. Sci. Instrum.* **1996**, *67*, 4072–4084.

[25] M. Melosso, A. Achilli, F. Tamassia, E. Canè, A. P. Charmet, P. Stoppa, L. Dore, *J. Quant. Spectrosc. Ra.* **2020**, 106982.

[26] M. Melosso, B. A. McGuire, F. Tamassia, C. Degli Esposti, L. Dore, *ACS Earth Space Chem.* **2019**, *3*, 1189–1195.

[27] C. Degli Esposti, M. Melosso, L. Bizzocchi, F. Tamassia, L. Dore, *J. Mol. Struct.* **2020**, *1203*, 127429.

[28] H. M. Pickett, *J. Mol. Spectrosc.* **1991**, *148*, 371–377.

[29] D. H. Hunter, S. Sim, *Can. J. Chem.* **1972**, *50*, 669–677.

[30] R. Kupfer, U. H. Brinker, *J. Org. Chem.* **1996**, *61*, 4185–4186.

[31] P. Młynarz, T. Ptak, A. Czernicka, R. Pankiewicz, K. Gluza, D. Zarzeczańska, *J. Mol. Struct.* **2011**, *991*, 18–23.

[32] I. Pecnikaj, F. Foschi, R. Bucci, M. L. Gelmi, C. Castellano, F. Meneghetti, M. Penso, *European J. Org. Chem.* **2019**, *2019*, 6707–6713.
